# Neurotrophic–Inflammatory Imbalance and Cognitive Decline After Adjuvant Chemotherapy in Colon Cancer: A Prospective Cohort Study

**DOI:** 10.1002/pon.70590

**Published:** 2026-08-25

**Authors:** Zeynep Gülsüm Güç, Leyla Demir, Hüseyin Döngelli, İbrahim Bilen, Mert Uge, Mehmet Eren Kalender, Ahmet Alacacıoğlu, Mustafa Oktay Tarhan

**Affiliations:** ^1^ Department of Medical Oncology Atatürk Training and Research Hospital İzmir Katip Çelebi University İzmir Türkiye; ^2^ Department of Biochemistry Atatürk Training and Research Hospital İzmir Katip Çelebi University İzmir Türkiye; ^3^ Department of Internal Medicine Buca Seyfi Demirsoy Training and Research Hospital İzmir Türkiye; ^4^ Department of Medical Oncology Sakarya Training and Research Hospital Sakarya Türkiye; ^5^ Department of Biochemistry Karabük Public Health Laboratory Karabük Türkiye; ^6^ Department of Medical Oncology Institute of Oncology Dokuz Eylül University İzmir Türkiye

**Keywords:** biomarkers, brain‐derived neurotrophic factor, cancer‐related cognitive impairment, colon cancer, inflammation, neuroplasticity, oxaliplatin, psycho‐oncology

## Abstract

**Background:**

Cancer‐related cognitive impairment (CRCI) is a prevalent yet underrecognized complication among colon cancer survivors that adversely affects quality of life and psychological well‐being. Emerging evidence suggests that neurotrophic dysregulation and systemic inflammation may contribute to chemotherapy‐related neurotoxicity; however, longitudinal biomarker‐based data remain limited.

**Methods:**

In this prospective cohort study, 127 patients with resected colon cancer were evaluated at baseline and at the 6‐month follow‐up. Of these, 83 received adjuvant chemotherapy and 44 underwent postoperative surveillance alone. Cognitive performance (MoCA), depressive symptoms (HADS‐D), and peripheral neuropathy (CIPN20) were assessed alongside serum brain‐derived neurotrophic factor (BDNF) levels and systemic inflammatory markers, including neutrophil‐to‐lymphocyte ratio (NLR) and C‐reactive protein‐to‐albumin ratio (CAR). Linear mixed model and logistic regression analyses were performed to identify factors associated with cognitive decline.

**Results:**

Adjuvant chemotherapy was associated with significant reductions in MoCA scores and serum BDNF levels (both *p* < 0.001), particularly among patients receiving the oxaliplatin‐based XELOX regimen. Greater longitudinal reductions in BDNF (ΔBDNF) were independently associated with higher odds of CRCI (OR = 0.749 per 10 ng/mL increase in ΔBDNF, 95% CI: 0.658–0.854, *p* < 0.001). The model including ΔBDNF showed higher apparent discrimination than the corresponding model without ΔBDNF (AUC = 0.929 vs. 0.812).

**Conclusion:**

Longitudinal reductions in serum BDNF were independently associated with CRCI among patients receiving adjuvant chemotherapy. These findings support a potential role for neurotrophic dysregulation in chemotherapy‐related cognitive decline and highlight the need for further validation studies evaluating BDNF as a candidate biomarker for CRCI.

## 1 Introduction

With advances in surgical and adjuvant therapies, an increasing number of colon cancer patients now live well beyond their initial diagnosis, bringing survivorship and post‐treatment quality of life to the forefront of oncology care [1]. Among late effects, cancer‐related cognitive impairment (CRCI) has gained growing recognition as a source of functional limitation and psychological distress, affecting attention, memory, and executive functioning, and consequently, patients' ability to reintegrate socially and professionally [2, 3]. Bibliometric analyses confirm that research interest in cognitive outcomes across cancer types has increased substantially over the past decade, yet the field remains methodologically heterogeneous and largely focused on breast cancer [4]. Although CRCI has been extensively studied in breast cancer survivors, data in patients with colon cancer—particularly those receiving fluoropyrimidine‐ and oxaliplatin‐based adjuvant regimens—remain limited [5, 6]. Notably, cognitive difficulties in colon cancer survivors may manifest across multiple domains and persist well beyond the completion of treatment, with implications for daily functioning and independent living [7, 8].

Despite its clinical relevance, the neurobiological mechanisms underlying CRCI remain poorly characterized. Neurotrophic and inflammatory signaling pathways are thought to play complementary roles in chemotherapy‐induced neurotoxicity. Brain‐derived neurotrophic factor (BDNF), a key mediator of synaptic plasticity, neurogenesis, and cognitive resilience, may be suppressed under chemotherapy‐induced oxidative and inflammatory stress, thereby compromising neuroprotective signaling [2, 9]. Converging preclinical and clinical evidence suggests that reductions in circulating BDNF parallel deteriorations in cognitive performance, particularly following cytotoxic treatment [10].

Systemic inflammation is increasingly recognized as a mechanistic contributor to CRCI. Among available inflammatory indices, the neutrophil‐to‐lymphocyte ratio (NLR) reflects the balance between innate immune activation and adaptive immune suppression, and has been associated with neuroinflammatory processes and poorer cognitive outcomes in cancer populations [11]. The C‐reactive protein‐to‐albumin ratio (CAR) integrates systemic inflammatory burden with nutritional status, offering a composite index of physiological stress that may amplify central neuroinflammatory cascades and further compromise BDNF–TrkB signaling [11]. Both markers are readily available from routine blood tests, making them practical candidates for biomarker‐based cognitive risk stratification.

Objective longitudinal assessments integrating cognitive, affective, and biological dimensions remain scarce in colon cancer, and the interplay between neurotrophic dysregulation and systemic inflammation in this setting has not been prospectively evaluated. We therefore hypothesized that adjuvant chemotherapy would induce concurrent reductions in serum BDNF and elevations in inflammatory markers, and that these changes would be independently associated with longitudinal cognitive decline.

The present prospective cohort study aimed to characterize dynamic changes in serum BDNF, NLR, and CAR among patients with colon cancer undergoing adjuvant chemotherapy and to evaluate their longitudinal associations with cognitive performance and CRCI.

## 2 Material and Methods

### 2.1 Study Design and Ethical Approval

This prospective observational cohort study investigated associations between neurotrophic (BDNF) and systemic inflammatory (NLR, CAR) biomarkers and cognitive performance in patients with resected colon cancer, including those receiving adjuvant chemotherapy and those undergoing postoperative surveillance alone. All participants were evaluated at baseline prior to initiation of any adjuvant treatment (T0) and again at the 6‐month follow‐up (T1). The study was conducted in accordance with the Declaration of Helsinki and approved by the local institutional ethics committee (Approval No: 2024‐GOKAE‐0294). Written informed consent was obtained from all participants.

### 2.2 Study Population

Patients aged ≥ 18 years with histopathologically confirmed colon adenocarcinoma who had undergone curative resection were enrolled. Patients were classified into two groups: those receiving adjuvant chemotherapy and those managed with postoperative surveillance alone based on favorable clinicopathological features. All participants were required to have an ECOG performance status of 0–1 and to be free from comorbidities potentially affecting cognitive function or systemic inflammation.

Exclusion criteria included rectal cancer, as well as pre‐existing inflammatory, neurological, or psychiatric disorders, metabolic causes of cognitive impairment (e.g., diabetes mellitus, vitamin B12 deficiency, hypothyroidism), use of antidepressants or neuroactive medications (e.g., gabapentin, pregabalin), acute infections or laboratory evidence of systemic inflammation at enrollment, metastatic disease, and incomplete adjuvant therapy.

### 2.3 Treatment Groups

Patients receiving adjuvant chemotherapy were treated with either single‐agent capecitabine or oxaliplatin‐based XELOX combination therapy, in accordance with current national and international oncology guidelines. Treatment allocation was determined by the treating oncologist based on clinicopathological risk assessment. Patients with favorable pathological features who did not meet criteria for adjuvant chemotherapy were managed with postoperative surveillance alone and served as a non‐chemotherapy comparator group.

### 2.4 Assessment Schedule and Measurements

Assessments were performed at two time points: baseline prior to treatment initiation (T0) and at the 6‐month follow‐up (T1). Cognitive function was evaluated using the Montreal Cognitive Assessment (MoCA). Serum BDNF levels were measured from fasting morning blood samples using enzyme‐linked immunosorbent assay (ELISA). Systemic inflammation was assessed using the neutrophil‐to‐lymphocyte ratio (NLR) and C‐reactive protein‐to‐albumin ratio (CAR), both derived from routine hematology and biochemistry parameters.

Depressive symptoms were evaluated using the Hospital Anxiety and Depression Scale–Depression subscale (HADS‐D), and chemotherapy‐induced peripheral neuropathy was assessed using the EORTC QLQ‐CIPN20 questionnaire. Demographic, clinical, and pathological data were recorded for all participants at enrollment.

### 2.5 Neurocognitive Assessment

The Montreal Cognitive Assessment (MoCA) assesses multiple cognitive domains, including memory, attention, executive function, language, visuospatial ability, and orientation, with a maximum score of 30 points. One additional point was added for participants with ≤ 12 years of formal education, as recommended in the original validation study. The MoCA has been validated for use in oncology populations. A decline of ≥ 2 points between baseline and follow‐up was defined as clinically significant cognitive deterioration and used to define CRCI, consistent with thresholds applied in previous CRCI research [12].

### 2.6 Biomarker Analysis

Blood samples were centrifuged within 10 minutes of collection, and serum aliquots were stored at −80°C until analysis. Serum BDNF concentrations were measured using a commercial human BDNF ELISA kit (SEA011HU, USCNK Life Science Inc., Wuhan, China). Measurements were performed in duplicate with a coefficient of variation below 10%. Baseline and follow‐up samples were analyzed simultaneously using the same kit lot to minimize inter‐assay variability. NLR and CAR values were derived from routine hematological and biochemical parameters. Sampling was postponed in cases with suspected acute infection at the time of assessment.

### 2.7 Psychiatric and Neuropathy Assessments

Depressive symptoms were evaluated using the Hospital Anxiety and Depression Scale–Depression subscale (HADS‐D), which has been validated in oncology settings and minimizes confounding from somatic symptoms. A score of > 7 was considered indicative of clinically significant depressive symptoms.

Peripheral neuropathy was assessed using the EORTC QLQ‐CIPN20 questionnaire, which evaluates sensory, motor, and autonomic symptoms. Both instruments were administered through face‐to‐face structured interviews at baseline (T0) and at the 6‐month follow‐up (T1).

### 2.8 Statistical Analysis

All statistical analyses were performed using IBM SPSS Statistics for Windows, Version 29.0 (IBM Corp., Armonk, NY, USA). Continuous variables were expressed as mean ± standard deviation (SD) for normally distributed data or median with interquartile range (IQR) for non‐normally distributed data, as appropriate. Categorical variables were presented as frequencies and percentages.

Normality of continuous variables was assessed using the Shapiro–Wilk test together with visual inspection of histograms. Between‐group comparisons were performed using the independent samples *t*‐test or Mann–Whitney *U* test for continuous variables, and the chi‐square test or Fisher's exact test for categorical variables, as appropriate.

Cancer‐related cognitive impairment (CRCI) was defined as a decline of ≥ 2 points in the Montreal Cognitive Assessment (MoCA) score from baseline to the 6‐month follow‐up, consistent with previously reported thresholds for clinically meaningful longitudinal cognitive change [12].

#### 2.8.1 Linear Mixed Model Analysis

To evaluate longitudinal associations between cognitive performance and clinical variables, linear mixed model (LMM) analyses were performed with MoCA score as the dependent variable. Several covariance structures were explored, including unstructured and autoregressive models. More complex covariance structures resulted in convergence instability and reduced model parsimony; therefore, the compound symmetry structure was selected based on model fit and parameter stability. Time (baseline and 6‐month follow‐up) was treated as a repeated measure, and model parameters were estimated using restricted maximum likelihood (REML). Type III F‐tests with Satterthwaite approximation were used to evaluate fixed effects. Clinical variables were entered as fixed effects, whereas the covariance structure was applied only to repeated MoCA measurements within subjects. A subject‐level random intercept was not included because models incorporating a random intercept resulted in model instability; therefore, within‐subject correlation was instead modeled using the compound symmetry covariance structure described above. Regression coefficients (*β*), standard errors (SE), 95% confidence intervals (CI), and *p*‐values were reported.

Primary analyses were conducted among patients receiving adjuvant chemotherapy (*n* = 83). The longitudinal dataset was structured in long format, with one observation per participant at each assessment time point. Time was modeled as a categorical variable representing baseline and the 6‐month follow‐up, with baseline serving as the reference category. BDNF was entered as a time‐varying continuous covariate, such that the BDNF concentration measured at each assessment was paired with the corresponding MoCA measurement rather than carrying the baseline BDNF value forward to follow‐up. BDNF was entered in its original units (ng/mL) without centering or standardization. The Time × BDNF interaction was included to assess whether the association between BDNF and cognitive performance differed between baseline and 6 months. Fixed effects included time, BDNF, the Time × BDNF interaction, chemotherapy regimen (capecitabine vs. capecitabine plus oxaliplatin), age, sex, educational level, and baseline depressive symptoms (HADS‐D; dichotomized as ≤ 7 vs. > 7). The final model was specified to include variables considered clinically relevant to the primary hypothesis while maintaining model parsimony. Exploratory analyses including the entire cohort (*n* = 127) are presented in the Supporting Information S1; fixed effects in that model additionally included a surveillance group (no adjuvant chemotherapy).

Among patients receiving adjuvant chemotherapy, pathological characteristics according to chemotherapy regimen (capecitabine vs. capecitabine plus oxaliplatin) were also compared. TNM stage, lymphatic invasion, vascular invasion, and perineural invasion were compared using the chi‐square test or Fisher's exact test, as appropriate. The number of involved lymph nodes was compared between treatment groups using an appropriate test according to data distribution. As chemotherapy regimen was allocated according to an institutional treatment algorithm based on pathological stage, chemotherapy regimen and pathological stage were highly collinear in this cohort; therefore, these variables were not simultaneously included in the same multivariable model. The additional pathological variables were not included as covariates in the primary LMM, which was maintained as a parsimonious model focused on the longitudinal biomarker–cognition associations. These comparisons are presented in Supporting Information S1: Table S3.

#### 2.8.2 Binary Logistic Regression Analysis

Binary logistic regression analyses were conducted among patients receiving adjuvant chemotherapy (*n* = 83) to identify factors associated with CRCI. Univariate analyses were initially performed for candidate variables including demographic, clinical, inflammatory, neuropsychological, and neurotrophic markers. Given the occurrence of 39 CRCI events among 83 patients, the final multivariable model was deliberately restricted to a parsimonious set of four variables to reduce the potential risk of model overfitting. The final model included sex, chemotherapy regimen, ΔBDNF, and baseline CAR. Although HADS‐D showed a borderline association in univariate analysis (*p* = 0.079), it was not retained in the final multivariable model because the model was intentionally restricted to a limited number of variables in view of the number of outcome events. Thus, the univariate *p*‐value threshold was used for exploratory assessment of candidate variables but was not applied as an automatic inclusion criterion for the final model. To minimize multicollinearity, highly correlated longitudinal biomarker measures (e.g., baseline, 6‐month, and change scores) were not simultaneously included in the same multivariable model. Odds ratios (ORs) with 95% confidence intervals (CIs) were reported.

Model calibration was evaluated using the Hosmer–Lemeshow goodness‐of‐fit test, whereas overall model fit was assessed using the Omnibus test and Nagelkerke R^2^ statistic.

#### 2.8.3 Receiver Operating Characteristic (ROC) Analysis

ROC curve analyses were performed to evaluate the discriminative performance of selected biomarkers and multivariable logistic regression models for CRCI among patients receiving adjuvant chemotherapy (*n* = 83). The area under the curve (AUC) with 95% confidence intervals was calculated for each analysis. Optimal cut‐off values were determined using the Youden index (sensitivity + specificity − 1). Sensitivity and specificity corresponding to the optimal thresholds were reported. For BDNF‐related variables, lower BDNF values were considered indicative of higher CRCI risk.

Given the modest sample size and absence of external validation, ROC findings were considered exploratory and interpreted cautiously.

A two‐tailed *p*‐value < 0.05 was considered statistically significant for all analyses.

## 3 Results

### 3.1 Patient and Tumor Characteristics

A total of 127 patients with curatively resected colon cancer were included. Of these, 83 (65.4%) received adjuvant chemotherapy (capecitabine or capecitabine plus oxaliplatin), whereas 44 (34.6%) were managed with postoperative surveillance alone. The mean age was 57.8 ± 10.5 years, and the majority of patients were male (59.8%).

Histopathologically, 90.6% of tumors were adenocarcinomas, 7.1% mucinous adenocarcinomas, and 2.4% signet‐ring cell carcinomas. Tumors were well differentiated in 59.8% of patients, moderately differentiated in 34.6%, and poorly differentiated in 4.7%. Lymphatic, vascular, and perineural invasion were observed in 30.7%, 4.7%, and 7.9% of cases, respectively.

Compared with the surveillance group, patients receiving adjuvant chemotherapy had significantly higher rates of lymphatic invasion (47.0% vs. 0%, *p* < 0.001), perineural invasion (12.0% vs. 0%, *p* = 0.015), lymph node metastasis (*p* < 0.001), and more advanced TNM stage distribution (*p* < 0.001). All stage I patients were managed with surveillance alone, whereas all stage III patients received adjuvant chemotherapy. The two groups were comparable in terms of age, sex, educational level, tumor localization, histopathological subtype, and histopathological grade (all *p* > 0.05), whereas they differed significantly in nodal involvement, lymphatic and perineural invasion, and TNM stage distribution, reflecting expected differences based on treatment indication.

Baseline demographic, clinicopathological, clinical, and biomarker characteristics are summarized in Table 1.

**TABLE 1:** Comparison of patient characteristics by adjuvant chemotherapy alongside overall population profiles.

	No adjuvant chemotherapy (*n*:44)	Adjuvant chemotherapy (*n*:83)	Total (*n*:127)	*p*
Age mean ± SD	56.1 ± 11.3	58.8 ± 9.9	57.8 ± 10.5	0.168
Sex *n* (%)				0.375
Male	24 (54.5)	52 (62.7)	76 (59.8)	
Female	20 (45.5)	31 (37.3)	51 (40.2)	
Educational level *n* (%)				0.198
No schooling	7 (15.9)	6 (7.2)	13 (10.2)	
Primary school	23 (52.3)	36 (43.4)	59 (46.5)	
High school	9 (20.5)	27 (32.5)	36 (28.3)	
University	5 (11.4)	14 (16.9)	19 (15.0)	
Localisation *n* (%)				0.504
Right side	25 (56.8)	42 (50.6)	67 (52.8)	
Left side	19 (43.2)	41 (49.4)	60 (47.2)	
Histopathological subtypes *n* (%)				0.716
Adenocarcinoma	41 (93.2)	74 (89.2)	115 (90.6)	
Mucinous adenocarcinoma	2 (4.5)	7 (8.4)	9 (7.1)	
Signet ring cell carcinoma	1 (2.3)	2 (2.4)	3 (2.4)	
Histopathological grade *n* (%)				0.558
Well differentiated	27 (61.4)	49 (59.0)	76 (59.8)	
Moderately differentiated	14 (31.8)	30 (36.1)	44 (34.6)	
Poorly differentiated	2 (4.5)	4 (4.8)	6 (4.7)	
Undifferentiated	1 (2.3)	0 (0)	1 (0.8)	
Number of lymph node excised median (IQR)	22 (15)	19 (12)	20 (14)	0.057
Number of lymph node involvement median (IQR)	1 (0)	1 (2)	1 (1)	**< 0.001**
Lymphatic invasion *n* (%)	0 (0)	39 (47.0)	39 (30.7)	**< 0.001**
Vascular invasion *n* (%)	0 (0)	6 (7.2)	6 (4.7)	0.092
Perineural invasion *n* (%)	0 (0)	10 (12.0)	10 (7.9)	**0.015**
TNM stage *n* (%)				**< 0.001**
1	20 (45.5)	0 (0)	20 (15.7)	
2	24 (54.5)	41 (49.4)	65 (51.2)	
3	0 (0)	42 (50.6)	42 (33.1)	
4	0 (0)	0 (0)	0 (0)	
Adjuvant chemotherapy *n* (%)				**< 0.001**
None	44 (100)	0 (0)	44 (34.6)	
Capecitabine	0 (0)	42 (50.6)	42 (33.1)	
Capecitabine + oxaliplatin	0 (0)	41 (49.4)	41 (32.3)	
BDNF baseline mean ± SD	206 ± 60	207 ± 50	207 ± 53	0.954
BDNF at 6th month mean ± SD	172 ± 65	116 ± 77	135 ± 76	**< 0.001**
NLR baseline mean ± SD	1.21 ± 0.49	1.41 ± 0.58	1.43 ± 0.57	**0.002**
NLR at 6th month mean ± SD	2.11 ± 0.76	3.34 ± 1.68	2.91 ± 1.54	**< 0.001**
CAR baseline median (IQR)	0.24 (0.02)	0.27 (0.02)	0.26 (0.02)	0.630
CAR at 6th month median (IQR)	0.54 (1.06)	2.10 (6.76)	1.09 (5.28)	**< 0.001**
HADS‐D baseline *n* (%)				0.217
0–7	42 (95.5)	72 (86.7)	114 (89.9)	
> 7	2 (4.5)	11 (13.3)	13 (10.2)	
HADS‐D at 6th month *n* (%)				**0.033**
0–7	38 (86.4)	57 (68.7)	95 (74.8)	
> 7	6 (13.6)	26 (31.3)	32 (25.2)	
CIPN20 baseline mean ± SD	61 ± 11	55 ± 7	57 ± 9	**0.002**
CIPN20 at 6th month mean ± SD	63 ± 11	67 ± 19	65 ± 17	0.130
MoCA baseline mean ± SD	27.3 ± 1.5	26.8 ± 1.5	26.9 ± 1.5	0.071
MoCA at 6th month mean ± SD	26.9 ± 1.5	24.9 ± 2.3	25.6 ± 2.2	**< 0.001**
CRCI *n* (%)	2 (4.5)	39 (47.0)	41 (32.3)	**< 0.001**

*Note:* Bold values indicate statistically significant results (*p* < 0.05).

Abbreviations: BDNF, brain‐derived neurotrophic factor; CAR, C‐reactive protein‐to‐albumin ratio; CIPN20, European Organisation for Research and Treatment of Cancer Quality of Life Questionnaire–Chemotherapy‐Induced Peripheral Neuropathy 20; CRCI, cancer‐related cognitive impairment; HADS‐D, Hospital Anxiety and Depression Scale–Depression subscale; IQR, interquartile range; MoCA, Montreal Cognitive Assessment; NLR, neutrophil‐to‐lymphocyte ratio; SD, standard deviation.

Among patients receiving adjuvant chemotherapy, clinicopathological characteristics differed substantially according to treatment regimen (Supporting Information S1: Table S3). Nearly all patients receiving capecitabine had stage II disease (97.6%), whereas all patients receiving XELOX had stage III disease (100%; *p* < 0.001). The number of involved lymph nodes was also higher in the XELOX group (median 2 vs. 0, *p* < 0.001). Lymphatic, vascular, and perineural invasion rates did not differ significantly between the two treatment groups.

### 3.2 Cognitive Performance, Serum BDNF, and Inflammatory Biomarkers

Baseline MoCA scores did not differ significantly between the surveillance and adjuvant chemotherapy groups (*p* = 0.071). At the 6th‐month follow‐up, patients receiving adjuvant chemotherapy demonstrated significantly lower MoCA scores compared with those under surveillance (24.9 ± 2.3 vs. 26.9 ± 1.5, *p* < 0.001).

Serum BDNF levels were comparable between groups at baseline (approximately 207 ± 53 ng/mL overall). At the 6th month, BDNF levels decreased markedly among patients receiving chemotherapy (116 ± 77 ng/mL), whereas only a modest, non‐significant decline was observed in the surveillance group (172 ± 65 ng/mL) (*p* < 0.001 for between‐group difference at 6th month; Table 1).

Systemic inflammatory markers also increased significantly following adjuvant chemotherapy. Mean NLR increased from 1.41 ± 0.58 to 3.34 ± 1.68, and median CAR from 0.27 to 2.10 (both *p* < 0.001). In contrast, changes in inflammatory markers were less pronounced in the surveillance group.

Depressive symptoms and peripheral neuropathy scores worsened modestly during follow‐up, with higher HADS‐D and CIPN20 scores observed among patients receiving adjuvant chemotherapy at the 6th month (Table 1).

### 3.3 Linear Mixed Model Analysis Among Patients Receiving Adjuvant Chemotherapy

Exploratory analyses involving the overall cohort are presented in the Supporting Information S1: Tables S1–S2.

Multivariable linear mixed model analysis among patients receiving adjuvant chemotherapy (*n* = 83) demonstrated a significant decline in cognitive performance at the 6th‐month follow‐up compared with baseline (*β* = −3.314, 95% CI: −4.309 to −2.319, *p* < 0.001).

Educational level was independently associated with cognitive outcomes; patients without formal education had significantly lower MoCA scores compared with university graduates (*β* = −2.817, 95% CI: −4.346 to −1.289, *p* < 0.001). Patients receiving capecitabine monotherapy demonstrated significantly higher MoCA scores compared with those treated with the XELOX regimen (*β* = 1.035, 95% CI: 0.354–1.716, *p* = 0.003), suggesting poorer cognitive performance among patients receiving oxaliplatin‐based treatment.

At baseline, serum BDNF levels were positively associated with MoCA performance (*β* = 0.018, 95% CI: 0.014–0.021, *p* < 0.001). The significant BDNF × Time interaction (*β* = −0.015, 95% CI: −0.020 to −0.010, *p* < 0.001) indicated that this association was attenuated at the 6‐month follow‐up.

No significant associations were observed between MoCA scores and age, sex, or baseline depressive symptoms (all *p* > 0.05). Detailed model estimates are presented in Table 2.

**TABLE 2:** Linear mixed model analysis of factors associated with MoCA scores among patients receiving adjuvant chemotherapy (*n* = 83).

Variables	β (95% CI)	SE	t	*p*
Age	−0.024 (−0.058/0.009)	0.017	−1.4	0.153
Female sex	0.183 (−0.498/0.864)	0.342	0.5	0.594
Time (6th month vs. baseline)	−3.314 (−4.309/‐2.319)	0.501	−6.6	**< 0.001**
Educational level (Reference: University)				
No education	−2.817 (−4.346/‐1.289)	0.769	−3.7	**< 0.001**
Primary school	−0.406 (−1.403/0.589)	0.500	−0.8	0.419
High school	−0.083 (−1.084/0.918)	0.503	−0.2	0.870
Adjuvant chemotherapy (capecitabine vs. XELOX)	1.035 (0.354/1.716)	0.342	3.0	**0.003**
Baseline depressive symptoms (HADS‐D > 7)	−0.253 (−0.778/0.272)	0.266	−0.9	0.342
BDNF	0.018 (0.014/0.021)	0.002	10.5	**< 0.001**
BDNFxTime (6‐month vs. baseline)	−0.015 (−0.020/‐0.010)	0.002	−6.1	**< 0.001**

*Note:* Bold values indicate statistically significant results (*p* < 0.05).

Abbreviations: BDNF, brain‐derived neurotrophic factor; HADS‐D, Hospital Anxiety and Depression Scale–Depression; MoCA, Montreal Cognitive Assessment; XELOX, capecitabine plus oxaliplatin.

Because baseline was specified as the reference category for Time, the main‐effect coefficient for BDNF represents the association between BDNF and MoCA at baseline, whereas the BDNF × Time interaction represents the change in this association at the 6‐month follow‐up. BDNF was nevertheless modeled as a time‐varying covariate, with each participant's BDNF measurement paired with the corresponding MoCA assessment.

### 3.4 Factors Associated With Cognitive Impairment (Logistic Regression Analysis)

Factors associated with CRCI among patients receiving adjuvant chemotherapy (*n* = 83) were evaluated using logistic regression analysis.

In univariate analyses, female sex (OR = 2.533, 95% CI: 1.016–6.315, *p* = 0.046), treatment with the XELOX regimen (OR = 6.070, 95% CI: 2.343–15.723, *p* < 0.001), greater reductions in BDNF over time (ΔBDNF per 10 ng/mL increase; OR = 0.730, 95% CI: 0.647–0.824, *p* < 0.001), and higher baseline CAR levels (OR = 1.920, 95% CI: 1.313–2.809, *p* = 0.001) were associated with an increased risk of CRCI.

In the multivariable model, only ΔBDNF remained independently associated with CRCI (OR = 0.749 per 10 ng/mL increase, 95% CI: 0.658–0.854, *p* < 0.001). Female sex, chemotherapy regimen, and baseline CAR did not retain statistical significance after adjustment.

Model calibration and overall fit were acceptable (Omnibus test *p* < 0.001; Hosmer–Lemeshow test *p* = 0.552; Nagelkerke R^2^ = 0.674). Detailed univariate and multivariable logistic regression results are presented in Table 3.

**TABLE 3:** Logistic regression analysis of factors for CRCI (*n* = 83).

Variables	Univariate			Multivariate		
	OR	95% CI	*p*	OR	95% CI	*p*
Age	1.011	0.968–1.057	0.612	—	—	—
Female sex	2.533	1.016–6.315	**0.046**	2.448	0.600–9.984	0.212
Chemotherapy (XELOX vs. Capecitabine)	6.070	2.343–15.723	**< 0.001**	2.302	0.601–8.835	0.224
ΔBDNF** (per 10 ng/mL increase)	0.730	0.647–0.824	**< 0.001**	0.749	0.658–0.854	**< 0.001**
ΔCIPN20	1.016	0.990–1.042	0.241	—	—	—
HADS‐D baseline	3.527	0.864–14.396	0.079	—	—	—
NLR baseline	1.193	0.563–2.528	0.644	—	—	—
CAR (100x) baseline	1.920	1.313–2.809	**0.001**	1.339	0.797–2.252	0.270

*Note:* Omnibus test, *p* < 0.001; Hosmer–Lemeshow test, *p* = 0.552; Nagelkerke R^2^ = 0.674. ΔBDNF was calculated as 6th‐month BDNF minus baseline BDNF. Bold values indicate statistically significant results (*p* < 0.05).

Abbreviations: BDNF, brain‐derived neurotrophic factor; CAR, C‐reactive protein‐to‐albumin ratio; CI, confidence interval; CIPN20, European Organisation for Research and Treatment of Cancer Quality of Life Questionnaire–Chemotherapy‐Induced Peripheral Neuropathy 20; CRCI, cancer‐related cognitive impairment; HADS‐D, Hospital Anxiety and Depression Scale–Depression subscale; NLR, neutrophil‐to‐lymphocyte ratio; OR, odds ratio; XELOX, capecitabine plus oxaliplatin.

### 3.5 Receiver Operating Characteristic (ROC) Analysis

ROC curve analysis demonstrated variable discriminative performance of biomarkers and multivariable models for identifying CRCI among patients receiving adjuvant chemotherapy.

Among individual biomarkers, 6‐month BDNF demonstrated the highest discriminative ability (AUC = 0.940, 95% CI: 0.893–0.987, *p* < 0.001), followed by ΔBDNF (AUC = 0.917, 95% CI: 0.858–0.976, *p* < 0.001), baseline CAR (AUC = 0.765, 95% CI: 0.660–0.870, *p* < 0.001), and baseline CIPN20 (AUC = 0.705, 95% CI: 0.595–0.816, *p* = 0.001). Baseline BDNF and baseline NLR did not demonstrate significant discriminative performance.

The multivariable logistic regression model including ΔBDNF demonstrated strong discriminative performance for CRCI (AUC = 0.929, 95% CI: 0.876–0.983, *p* < 0.001), with an optimal cut‐off of ≥ 0.49 yielding 87% sensitivity and 88% specificity. Exclusion of ΔBDNF reduced the AUC to 0.812 (95% CI: 0.719–0.905), indicating the incremental discriminative value of longitudinal BDNF change.

**FIGURE 1:**
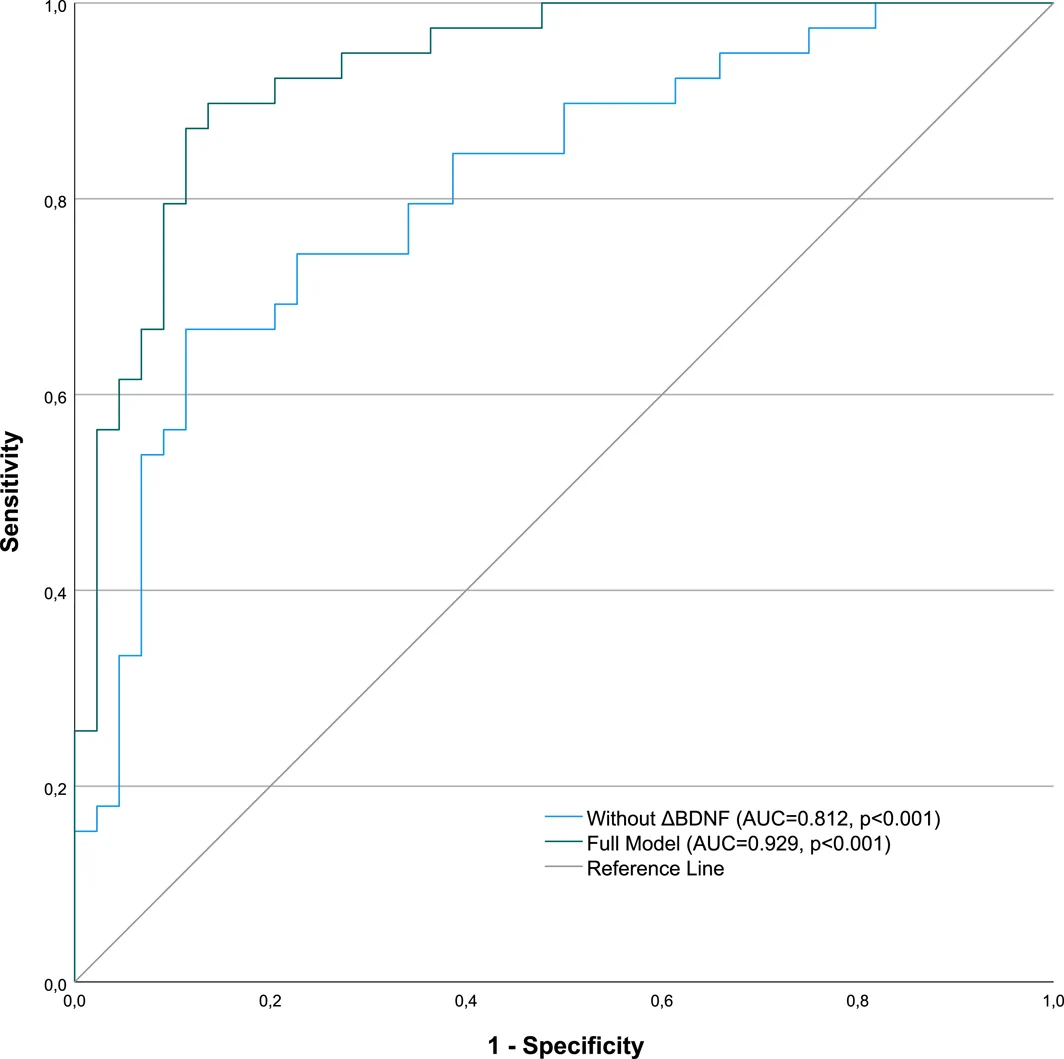
Receiver operating characteristic (ROC) curves of multivariable logistic regression models for classifying cancer‐related cognitive impairment (CRCI) among patients receiving adjuvant chemotherapy.

Optimal cut‐off values were determined using the Youden index. Detailed ROC metrics are summarized in Table 4, and the corresponding ROC curves are presented in Figure 1.

**TABLE 4:** Assessment of CRCI using ROC analysis of related factors and predicted probabilities of binary logistic regression models (*n* = 83).

Variables	CRCI			Sensitivity	Specificity
	AUC (95% CI)	*p*	Cut‐off		
BDNF baseline ng/mL	0.472 (0.347–0.598)	0.665	≤ 195.5	64%	48%
BDNF at 6th month ng/mL	0.940 (0.893–0.987)	**< 0.001**	≤ 95.0	75%	75%
ΔBDNF ng/mL	0.917 (0.858–0.976)	**< 0.001**	≤ −123.9	96%	67%
CIPN20 baseline	0.705 (0.595–0.816)	**0.001**	≥ 49.5	77%	93%
CIPN20 at 6th month	0.514 (0.387–0.641)	0.830	≥ 65.0	49%	68%
ΔCIPN20	0.621 (0.500–0.743)	0.058	≥ 3.7	54%	68%
NLR baseline	0.542 (0.417–0.667)	0.511	≥ 1.1	82%	36%
CAR baseline	0.765 (0.660–0.870)	**< 0.001**	≥ 0.0247	85%	64%
Final multivariable model	0.929 (0.876–0.983)	**< 0.001**	≥ 0.49	87%	88%
Multivariable model excluding ΔBDNF	0.812 (0.719–0.905)	**< 0.001**	≥ 0.50	74%	77%

*Note:* Cut‐off values were determined using the Youden index. For BDNF‐related variables, the “positive if ≤” direction was used so that lower BDNF values corresponded to higher CRCI risk. Bold values indicate statistically significant results (*p* < 0.05).

Abbreviations: AUC, area under the curve; BDNF, brain‐derived neurotrophic factor; CAR, C‐reactive protein‐to‐albumin ratio; CI, confidence interval; CIPN20, European Organisation for Research and Treatment of Cancer Quality of Life Questionnaire–Chemotherapy‐Induced Peripheral Neuropathy 20; CRCI, cancer‐related cognitive impairment; NLR, neutrophil‐to‐lymphocyte ratio.

The green curve represents the final multivariable model including ΔBDNF (AUC = 0.929, *p* < 0.001), whereas the blue curve represents the same model excluding ΔBDNF (AUC = 0.812, *p* < 0.001).

### 3.6 Longitudinal BDNF Trajectories According to CRCI Status

**FIGURE 2:**
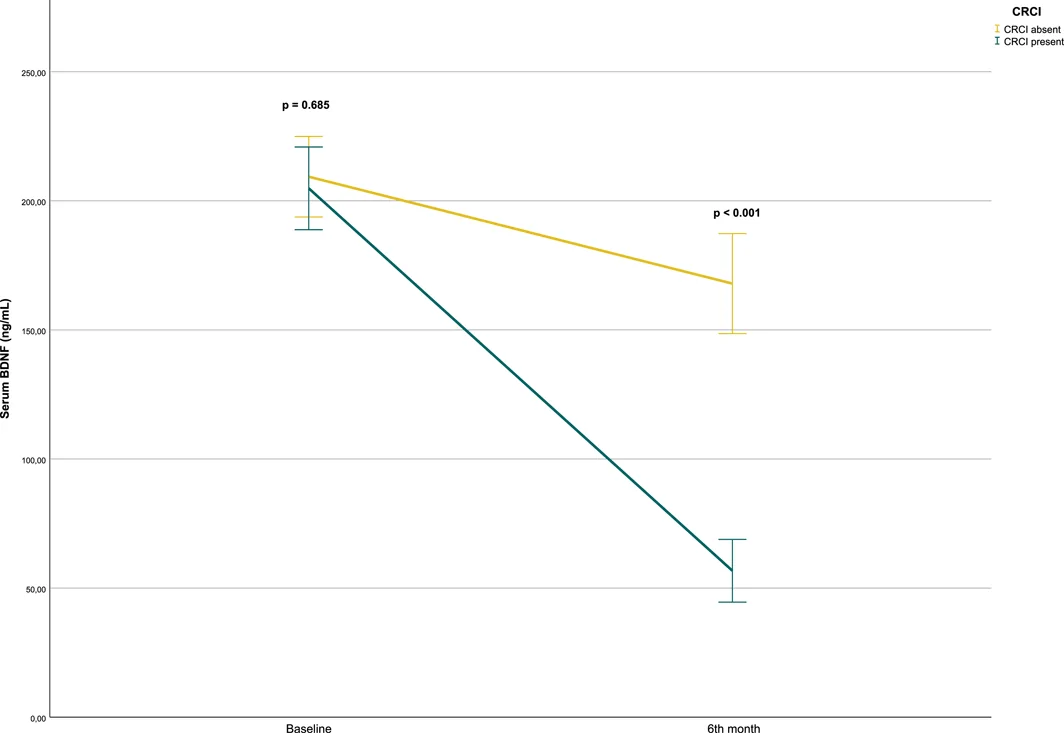
Mean serum BDNF levels at baseline and at the 6‐month follow‐up according to cancer‐related cognitive impairment (CRCI) status among patients receiving adjuvant chemotherapy. Patients who developed CRCI demonstrated a significantly greater longitudinal decline in BDNF levels over time compared with those without CRCI.

Longitudinal serum BDNF trajectories according to CRCI status are presented in Figure 2. At baseline, BDNF levels were comparable between patients who developed CRCI and those who did not (*p* = 0.685). At the 6‐month follow‐up, however, patients who developed CRCI demonstrated a significantly greater decline in BDNF levels compared with those without CRCI (*p* < 0.001).

## 4 Discussion

This prospective cohort study evaluated longitudinal associations between neurotrophic and inflammatory biomarkers and cognitive performance in patients with colon cancer receiving adjuvant chemotherapy following curative resection. Lower serum BDNF levels were associated with poorer cognitive performance, and the strength of this association differed between baseline and the 6‐month follow‐up. Although systemic inflammatory markers, including NLR and CAR, increased significantly during adjuvant chemotherapy, they did not retain independent associations with cognitive outcomes in multivariable analyses. Postoperative cognitive dysfunction has similarly been reported in elderly patients undergoing cancer surgery, suggesting that both surgical and chemotherapy‐related factors may contribute to long‐term neurocognitive sequelae in cancer survivors [13]. Together, these findings support a potential role for neurotrophic dysregulation in CRCI, whereas the independent contribution of systemic inflammation remains uncertain.

ROC analyses demonstrated favorable discriminative performance of BDNF‐based measures for identifying CRCI among patients receiving adjuvant chemotherapy. Both 6‐month BDNF levels (AUC = 0.940) and ΔBDNF (AUC = 0.917) showed the highest discriminative performance among the evaluated biomarkers. Incorporating ΔBDNF into the multivariable model resulted in a higher AUC than the corresponding model without ΔBDNF (0.929 vs. 0.812), suggesting additional discriminative information associated with longitudinal BDNF change. The optimal ΔBDNF cut‐off identified by the Youden index (≤−123.9 ng/mL) may warrant further investigation as a candidate threshold in future validation studies.

It should be noted that serum BDNF measurement is not yet part of routine clinical practice in oncology settings. Its implementation would require standardized assay protocols and prospective validation across diverse patient populations before clinical adoption could be recommended.

In the multivariable logistic regression model, ΔBDNF remained the only variable independently associated with CRCI after adjustment for sex, chemotherapy regimen, and baseline CAR (Table 3). Each 10 ng/mL reduction in BDNF was associated with higher CRCI risk. Although HADS‐D showed a borderline univariate association (*p* = 0.079), it was not retained in the final parsimonious multivariable model, which was restricted to four variables given the number of CRCI events (39 among 83 patients). The observed association between lower BDNF levels and poorer cognitive performance, together with the association between ΔBDNF and CRCI, supports a potential relationship between neurotrophic dysregulation and chemotherapy‐related cognitive decline.

These findings are consistent with previous studies in breast cancer survivors reporting parallel reductions in BDNF levels and cognitive performance [14, 15] while extending this evidence to patients with resected colon cancer receiving adjuvant chemotherapy. Together, these results support the hypothesis that neurotrophic alterations may contribute to CRCI, although residual confounding by depressive symptoms or peripheral neuropathy cannot be excluded.

Chemotherapy‐related neurotoxicity is considered a multifactorial process involving oxidative stress, inflammatory activation, and impaired neurotrophic signaling. Platinum‐based agents such as oxaliplatin have been associated with mitochondrial dysfunction, increased oxidative stress, and reductions in BDNF expression in preclinical studies [16, 17, 18, 19]. In the present cohort, patients treated with the XELOX regimen demonstrated greater cognitive decline and more pronounced reductions in BDNF. However, because chemotherapy regimen was closely linked to pathological stage in this cohort, these differences cannot be attributed specifically to oxaliplatin exposure. Larger studies with adequate representation of different pathological stages within each treatment regimen are needed to disentangle treatment‐ and disease‐related effects.

Previous studies have reported associations between reduced BDNF levels, inflammatory activation, and impaired cognitive performance following chemotherapy exposure [11]. In addition, a recent systematic review by Fierro‐Salgado et al. identified reduced hippocampal BDNF expression together with elevated pro‐inflammatory cytokines as potential biological correlates of CRCI [10].

Consistent with these observations, greater longitudinal reductions in serum BDNF were independently associated with cognitive decline in our cohort. Together, these findings support a potential contribution of neurotrophic dysregulation to the development of CRCI in patients with colon cancer receiving adjuvant chemotherapy.

Higher educational level was independently associated with better cognitive performance in the multivariable model, consistent with the concept of cognitive reserve. Prior evidence suggests that cognitive engagement and mental stimulation may enhance neurotrophic signaling and support resilience against chemotherapy‐related cognitive decline [20]. Neuropsychological assessments across different cancer types have similarly documented associations between educational attainment, post‐chemotherapy cognitive outcomes, and rehabilitation needs [21]. Although the present study was not designed to directly examine the relationship between educational level and BDNF trajectories, the observed association between lower education and greater cognitive decline is consistent with this framework. Lifestyle‐based approaches that promote cognitive engagement, such as physical exercise, social participation, and cognitive training, may help support cognitive resilience during adjuvant chemotherapy.

Depressive symptoms (HADS‐D) and peripheral neuropathy (CIPN20) are recognized contributors to cognitive complaints in patients receiving chemotherapy. In the present study, HADS‐D showed a borderline univariate association with CRCI (*p* = 0.079) but was not included in the final multivariable model, which was intentionally restricted to a limited number of variables given the number of outcome events; CIPN20 was not associated with CRCI in univariate analysis. Consequently, we cannot fully exclude the possibility that depressive symptoms or peripheral neuropathy partly confound the observed association between ΔBDNF and cognitive decline, and this should be regarded as a limitation. Nonetheless, the consistency of the ΔBDNF–CRCI association across the LMM and logistic regression analyses provides support for an association between longitudinal BDNF reductions and cognitive decline, although residual confounding cannot be excluded.

Together, these observations support the multifactorial nature of CRCI and highlight the importance of considering both biological and psychosocial factors when evaluating cognitive outcomes during chemotherapy.

These findings suggest that longitudinal monitoring of serum BDNF may provide additional clinical insight for identifying patients at higher risk of cognitive decline during adjuvant chemotherapy. Non‐pharmacological interventions associated with increases in circulating BDNF levels, such as structured physical exercise, cognitive training, and stress management, may represent supportive strategies for patients at risk of CRCI, although prospective intervention studies in colon cancer populations are needed to confirm their efficacy [22, 23, 24, 25]. Given the multifactorial nature of CRCI, integrative approaches addressing both biological and psychosocial dimensions of cognitive health may be beneficial in supportive cancer care.

### 4.1 Strengths

The principal strengths of this study include its prospective design, the inclusion of a non‐chemotherapy surveillance group as a comparator, and the homogeneity of the cohort achieved through systematic exclusion of patients with pre‐existing neurological, psychiatric, or inflammatory comorbidities. The concurrent and longitudinal assessment of serum BDNF alongside systemic inflammatory markers enabled evaluation of neurotrophic and inflammatory changes during adjuvant chemotherapy. The use of a validated objective cognitive measure (MoCA), together with the simultaneous assessment of depressive symptoms and peripheral neuropathy, further enhanced the interpretability of the findings.

### 4.2 Limitations

Several limitations should be acknowledged. First, cognitive assessment was limited to the Montreal Cognitive Assessment (MoCA), a screening instrument that may not fully capture subtle or domain‐specific cognitive deficits. In addition, the same MoCA version was administered at both assessment time points to maintain consistency across evaluations; therefore, potential practice effects cannot be entirely excluded. Second, BDNF was measured in peripheral serum without distinguishing between mature BDNF and pro BDNF isoforms. Although peripheral BDNF levels have been reported to correlate with central neurotrophic activity [26], serum measurements may not fully reflect central nervous system processes. Third, the single‐center design and relatively short follow‐up period may limit the generalizability of the findings and preclude assessment of longer‐term cognitive trajectories. In addition, chemotherapy regimen was strongly associated with pathological stage in this cohort, with capecitabine predominantly administered to patients with stage II disease and XELOX to patients with stage III disease. This close relationship limits our ability to distinguish treatment‐related effects from disease‐ or stage‐related differences in cognitive outcomes. Finally, the modest sample size, particularly within subgroup analyses, warrants cautious interpretation of the results. External validation in larger multicenter cohorts using comprehensive neuropsychological batteries is needed to confirm the reproducibility and clinical relevance of these findings.

### 4.3 Clinical Implications

The results of this study suggest that longitudinal monitoring of serum BDNF may provide additional insight into identifying patients at higher risk of cognitive decline during adjuvant chemotherapy for colon cancer. If validated in larger prospective cohorts, ΔBDNF may help identify patients who could benefit from supportive care services, including cognitive rehabilitation and psychological support interventions. These findings also highlight the potential relevance of incorporating neurotrophic and inflammatory biomarkers into future survivorship research frameworks.

## 5 Conclusion

In conclusion, longitudinal reductions in serum BDNF were independently associated with cognitive decline in patients with colon cancer receiving adjuvant chemotherapy. Although systemic inflammatory markers increased significantly during chemotherapy, they did not remain independently associated with cognitive outcomes after multivariable adjustment. These findings support a potential role for neurotrophic dysregulation in chemotherapy‐related cognitive decline, while the independent contribution of systemic inflammation remains uncertain and warrants further investigation.

Although further validation is required, these findings suggest that longitudinal biomarker assessment may contribute to future survivorship research and supportive care strategies aimed at improving cognitive outcomes in cancer survivors.

## Author Contributions

Conceptualization: Z.G.G., A.A., M.O.T. Methodology: Z.G.G., L.D.; Formal Analysis: Z.G.G., H.D., I.B. Investigation: Z.G.G., L.D., M.E.K. Writing – Original Draft: Z.G.G. Writing – Review & Editing: A.A., M.O.T. Supervision: A.A., M.O.T.

## Funding

This research was funded by the İzmir Oncology Group (İZOG) within the framework of independent academic support for translational cancer research. The funding organization had no role in the design, data collection, analysis, interpretation, or writing of the manuscript.

## Consent

Informed consent was obtained from all subjects included in the study.

## Conflicts of Interest

The authors declare no conflicts of interest.

## Institutional Review Board Statement

The study was conducted in accordance with the Declaration of Helsinki and approved by the Ethics Committee of İzmir Katip Çelebi University Atatürk Training and Research Hospital (approval number: [2024‐GOKAE‐0294], approval date: [29.7.2024]).

## Supporting information


Supporting Information S1

## Data Availability

The data that support the findings of this study are available from the corresponding author upon reasonable request.
