# Supplementary material for: Neurotrophic–Inflammatory Imbalance and Cognitive Decline After Adjuvant Chemotherapy in Colon Cancer: A Prospective Cohort Study
Source: Psychooncology. 2026 Aug 25;35(8):e70590. doi: 10.1002/pon.70590 (PMC13505728; doi:10.1002/pon.70590)
Supplement: Supplementary file 1 [file PON-35-e70590-s001.docx]

**Supplementary Table S1**. Linear mixed model analysis of factors associated with MoCA scores in the overall study cohort (n=127).

| **Variables** | **β (95% CI)** | **SE** | **t** | ***p*** |
| --- | --- | --- | --- | --- |
| Age | -0.018 (-0.044/0.007) | 0.013 | -1.4 | 0.166 |
| Female sex | 0.043 (-0.501/0.587) | 0.275 | 0.2 | 0.877 |
| Time (6th month vs baseline) | -2.328 (-3.022/-1.634) | 0.351 | -6.6 | **<0.001** |
| Educational level (Reference: University)  No education  Primary school  High school | -2.391 (-3.509/-1.271)  -0.537 (-1.352/0.278)  -0.079 (-0.915/0.757) | 0.566  0.412  0.422 | -4.2  -1.3  -0.2 | **<0.001**  0.194  0.852 |
| Adjuvant chemotherapy (Reference: XELOX)  None  Capecitabine | 1.421 (0.759/2.082)  1.071 (0.401/1.741) | 0.334  0.338 | 4.3  3.2 | **<0.001**  **0.002** |
| Baseline depressive symptoms (HADS-D >7) | -0.416 (-0.846/0.013) | 0.217 | -1.9 | 0.057 |
| BDNF | 0.016 (0.014/0.019) | 0.001 | 13.1 | **<0.001** |
| BDNF*Time (baseline vs 6th month) | -0.011 (-0.014/-0.007) | 0.002 | -6.2 | **<0.001** |

**Abbreviations:** BDNF, brain-derived neurotrophic factor; HADS-D, Hospital Anxiety and Depression Scale–Depression; MoCA, Montreal Cognitive Assessment; XELOX, capecitabine plus oxaliplatin.

**Supplementary Table S2.** Assessment of CRCI using ROC analysis of related factors and predicted probabilities of binary logistic regression models (n=127).

|  | **CRCI** | | |  |  |
| --- | --- | --- | --- | --- | --- |
| **Variables** | **AUC (95% CI)** | ***p*** | **Cut-off** | **Sensitivity** | **Specificity** |
| BDNF baseline *ng/mL* | 0.510 (0.407-0.614) | 0.851 | ≤268.8 | 20% | 97% |
| BDNF at 6th month *ng/mL* | 0.930 (0.884-0.976) | **<0.001** | ≤ 79.8 | 98% | 73% |
| ΔBDNF *ng/mL* | 0.925 (0.881-0.969) | **<0.001** | ≤ −86.2 | 86% | 90% |
| CIPN20 baseline | 0.753 (0.667-0.838) | **<0.001** | ≤ 59.0 | 50% | 88% |
| CIPN20 at 6th month | 0.492 (0.380-0.604) | 0.883 | ≤ 57.2 | 63% | 46% |
| ΔCIPN20 | 0.704 (0.608-0.800) | **<0.001** | ≥ 1.9 | 73% | 64% |
| NLR baseline | 0.654 (0.559-0.750) | **0.005** | ≥ 1.1 | 83% | 43% |
| CAR baseline | 0.718 (0.625-0.811) | **<0.001** | ≥ 0.0247 | 83% | 59% |
| Final multivariable model | 0.950 (0.912-0.988) | **<0.001** | ≥ 0.39 | 90% | 89% |
| Multivariable model excluding ΔBDNF | 0.891 (0.833-0.949) | **<0.001** | ≥ 0.35 | 83% | 81% |

**Abbreviations:** AUC, area under the curve; BDNF, brain-derived neurotrophic factor; CAR, C-reactive protein-to-albumin ratio; CI, confidence interval; CIPN20, European Organisation for Research and Treatment of Cancer Quality of Life Questionnaire–Chemotherapy-Induced Peripheral Neuropathy 20; CRCI, cancer-related cognitive impairment; NLR, neutrophil-to-lymphocyte ratio.

Cut-off values were determined using the Youden index. For BDNF-related variables, lower BDNF values were considered indicative of higher CRCI risk.

**Supplementary Table S3.** Clinicopathological characteristics according to adjuvant chemotherapy regimen among patients receiving chemotherapy (n=83)

| **Variable** | **Capecitabine (n=42)** | **XELOX (n=41)** | **p** |
| --- | --- | --- | --- |
| Stage, n (%)  II  III | 41 (97.6)  1 (2.4) | 0 (0)  41 (100) | **<0.001** |
| Number of lymph nodes involved, median (IQR) | 0 (0–0) | 2 (1–3) | **<0.001** |
| Lymphatic invasion *n (%)* | 16 (38.1) | 23 (56.1) | 0.100 |
| Vascular invasion *n (%)* | 4 (9.5) | 2 (4.9) | 0.676* |
| Perineural invasion *n (%)* | 4 (9.5) | 6 (14.6) | 0.520* |

* Fisher's exact test.

**Supplementary Figure S1.** ROC curve for multivariable model including ΔBDNF in the overall cohort

**Supplementary Figure S2.** ROC curve for multivariable model excluding ΔBDNF in the overall cohort
